# The “*Journal of Functional Morphology and Kinesiology*” Journal Club Series: Utility and Advantages of the Eccentric Training through the Isoinertial System

**DOI:** 10.3390/jfmk5010006

**Published:** 2020-01-19

**Authors:** James P. Fisher, Silvia Ravalli, Luke Carlson, Lee A. Bridgeman, Federico Roggio, Simone Scuderi, Mario Maniaci, Cristina Cortis, Andrea Fusco, Giuseppe Musumeci

**Affiliations:** 1 School of Sport, Health and Social Science, Solent University, Southampton SO14 0YN, UK; 2 Department of Biomedical and Biotechnological Sciences, Anatomy, Histology and Movement Sciences Section, School of Medicine, University of Catania, Via S. Sofia 87, 95123 Catania, Italy; 3 Discover Strength, 10160 6th Avenue North, Suite A, Plymouth, MN 55441, USA; 4 Department of Sport and Exercise Science, Solent University, East Park Terrace, Southampton SO14 0YN, UK; 5 Research Center on Motor Activities (CRAM), University of Catania, 95123 Catania, Italy; 6 Department of Human Sciences, Society and Health, University of Cassino e Lazio Meridionale, 03043 Cassino, Italy

## Abstract

We are glad to introduce the first Journal Club of volume five, the first issue. This edition is focused on relevant studies published in the last years in the field of eccentric training, chosen by our editorial board members and their colleagues. We hope to stimulate your curiosity in this field and to share with you the passion for the sport, seen also from a scientific point of view. The editorial board members wish you an inspiring lecture.

## 1. Introduction

Isoinertial technology was conceived as a countermeasure to the anatomic–physiological changes that the body undergoes during the permanence in microgravity. Initially, these machines were developed from the Swedish company Yoyo Technology, for astronauts orbiting around the Earth on board the ISS [1]. Outside the terrestrial atmosphere, in fact, it is essential to use isoinertial machines that are able to generate resistance regardless of gravity and weight [2]. Insoiertial machines generate resistance by putting a mass in rotation, by using one or two disks or fly-wheels (Yoyo technology), or a cord winded on a conic shaft (VersaPulley or conical pulley) [3]. A strap, wound on the shaft, is pulled during the concentric phase (CON) exercise and accelerates the flywheel, storing kinetic energy. The length of the rope is regulated to be completely untied at the end of the CON phase in the moment in which the fly wheel, continuing its rotation, re-wraps the rope. The subject, therefore, opposes an eccentric braking force (ECC), which slows it down until the complete arrest, with the rope being wrapped again, beginning the next repetition. The characteristic of isoinertial machines resides in the resistance, which is purely proportional to the acceleration—at a steady speed, there is no resistance, so the isoenertial exercise is conducted in a continual acceleration/deceleration. Furthermore, the resistance is proportional to the fly-wheel inertia, which depends on the diameter, as follows: if doubled, the inertia quadruples [4]. These machines allow the subject to perform tailored repetition—as the strength decreases with fatigue, the speed of the flywheel will consequently adapt by slowing. Therefore, the concept of a one-repetition maximum (1RM) does not exist in isoinertial devices, because the exercise, in a virtual way, could endlessly continue, even with significant inertia and very small forces. On the contrary, training with traditional weights requires an equal force in every repetition, and only the last one is by definition a 1RM [5]. Isoinertial technology is a valuable and innovative tool for speeding up the recovery times and improving the physical performance, which limits injuries through a superior eccentric overload compared with any other previous method. By generating high eccentric loads and reducing the moment of inertia, it is possible to improve the speed and muscle strength. This methodology reduces the incidence of injuries of the muscles of the back thigh, especially the biceps femoris (the muscle with the highest rate of injury). It is therefore imperative to promote a greater integration of these machines within the sports sectors, both in the rehabilitation and preventive fields, as they generate optimal stimuli at the musculoskeletal level, and represent the answer to the ever-increasing demand for the improvement of physical performance, without neglecting the recovery and prevention of accidents, in the shortest possible time.

## 2. Applicability in Sports: Soccer Case

### Highlight by Simone Scuderi and Mario Maniaci

The discipline in which the isoinertial concept is mainly used is football. Compared with other team sport, football has the highest percentage of injuries, most of them located in the lower limbs, specifically in the quadriceps, hamstrings, calfs, and adductors. Because of their features, the muscles of front and back thigh are seriously at risk of injuries for movements like a fast change of direction, kicks, or stops [6]. The amount of injuries could be reduced through eccentric training, because it allows for increasing the muscle lengthening, as the injury occurs when the activated muscle, during the contraction, lengthens beyond its optimal limit [7]. Askling et al. tested the use of the YoYo leg curl isoinertial machine on players of a Swedish premiere league team. For ten weeks, half of the team’s players included sessions of eccentric training in their pre-season program. After the season, the sum of the hamstring muscles injuries reported were only 3/15 in the group subjected to eccentric conditioning, against 10/15 of the other group. The authors suggest including this approach in daily soccer players’ training, both for injury prevention and for performance enhancement [8]. Furthermore, it has been noted by Hoyo et al., how, by subjecting athletes to a program that includes YoYo squats and leg curls, there is a decrease in the number of days of injury and recovery, and a lower percentage of overall lower limb injuries. Considering this evidence, it can be deduced that training through isoinertial devices is equivalent or even more effective than classical conditioning (e.g., weight lifting), improving both muscle size and strength, as well as recovery from hamstring injury [9].

## 3. Role in Rehabilitation from Injuries

### Highlight by Giuseppe Musumeci 

Skeletal muscle is a particular tissue that modifies its general functional ability in reaction to varied stimuli. The influence of the gravitational load on muscular performance is very important. Ordinarily, the daily action of gravity is enough to enable muscles to maintain their performance capabilities. However, with less gravitational load (microgravity), we have a clear reduction in both muscle mass and force-generating skill. Conversely, a growth in gravitational load (hypergravity) will increase in the cross-sectional area and force-generating aptitude of muscles. Exercise programs planned to increase strength and power are considered by performing exercises with an increase in gravitational load [10]. During muscle stretching in high speed activities, the eccentric phase requires a lower metabolic cost for the same effort compared with the concentric phase, and this involves less production of lactic acid, which means less perception of fatigue, which favors a longer working session. These features make eccentric training and the correlated isoinertial devices useful tools for recovery from injuries, through an action aimed at strengthening muscles and tendons with a protective action on the connective tissue. Brasileiro et al. [11] analyzed the effect of eccentric exercise during the rehabilitation of anterior cruciate ligament (ACL) injuries in sedentary subjects. The rationale behind this study relies on the assumption that eccentric training increases muscle trophism better than concentric exercise, as it involves higher protein synthesis stimulation and the recruitment of type II fibers. The eccentric training, administered to the patients, results in significantly increasing the proximal quadriceps’ region trophism, within 12 weeks. The authors suggest that strength improvement, as well as increases in the muscle cross-sectional area and contractile capacity, noted during the initial phase of training, would result from neuromuscular activation in combination with trophic factors. The late phase would be characterized, instead, by the most crucial activity of the trophic factors. Rehabilitation procedures, which aim at including isoinertial exercise, should take into consideration the nature of muscle deficiency, as well as post-traumatic difficulties related to pain or inflammatory processes. The latter suggests that exercise should be considered a fundamental point in the treatment of pathological skeletal muscle mass reduction [12].

## 4. Nutraceutical Supplementation in Isoinertial Performance

### Highlight by Silvia Ravalli 

Athletic improvements benefit from nutritional supplements, like creatine or β-alanine, in terms of their ergogenic effect. Among all, caffeine has a renowned resistance-induced property, involving Na^+^–K^+^ pump responses and calcium release mechanisms. Castillo et al. evaluated the effects of caffeine on lower limb power during a flywheel half-squat exercise on young, healthy, and active men [13]. This study suggests that caffeine supplementation (3–6 mg∙kg^−1^) induces improved power performances in exercises that in particular require resistance. Supplementation has also been proposed to avoid eccentric exercise-induced muscle damage, resulting from the breaking down of contractile components and inflammatory involvement in cases of excessive muscle tissue stress. Quercetin, a natural flavonoid, has been observed to exert a preventive effect on strength loss, muscle weakness, and electromyographic modifications that could follow eccentric exercise [14]. Its mechanism of action is supposed to involve membranes crossing and the stabilization of the excitation–contraction coupling in myocytes, preserving the contractile function, especially of Type II fibers, which are the ones maily involved in eccentric contractions. Another study by Tanabe et al. [15] supports the use of a nutraceutical compound, curcumin, in order to overcome the possible side effects of high-intensity eccentric exercise and faster recovery in case of muscle damage. Curcumin supplementation, after exercise, was shown to improve muscle strength, range of motion, pain, and serum creatine kinase activity, while curcumin supplementation before exercise attenuated the early inflammatory makers (IL-8). On the contrary, the high-fat western diet could impair muscle and liver metabolism, and lay the ground for subsequent muscle damage [16]. VitD, associated with a Mediterranean diet, showed a trophic action on the muscle fibers [17]. Physical exercise induces oxidative stress through the production of reactive oxygen species and can cause damage to the muscle tissue. Oxidative stress resulting from exhaustive exercise is high, and the improvement of the antioxidant defences of the body may ameliorate the damage caused by free radicals. Extra-virgin olive oil and physical activity is widely considered to possess anti-oxidative properties in skeletal muscles atrophy. The results of some studies support the view that extra-virgin olive oil can improve the adaptive response of the muscles in conditions of oxidative stress [18,19].

## 5. Use of Eccentric Training in Systemic Dysfunctions

### Highlight by Federico Roggio

Clinical practice benefits from the advantages of eccentric exercise in the management of chronic conditions, different from those that interest the musculoskeletal system or concern the world of sport performance. This type of exercise offers the convenience of being suited for those patients that would benefit from exercise, but present some difficulties that do not allow them to perform strenuous activities, like elders or those that suffer, for example, from neurological of cardiorespiratory conditions [20]. As eccentric training, despite concentric training, does not seem to modify arterial stiffness, it can be more easily prescribed to patients with coronary disease. Attenuated cardiopulmonary stress is another advantage, especially for elders or those ones not used to exercise. Eccentric resistance training has been shown to ameliorate functional task performance in subjects with Parkinson’s disease or neurological impairments, such as those related to stroke [20], or to improve pain management in patients with reduced functional mobility due to skeletal disorders, like osteoarthritis [21,22]. The treatment and prevention of metabolic diseases are also of interest for this training approach, which is better than concentric exercise at improving glucose tolerance and inducing favourable changes in the blood lipid profile [23]. Eccentric exercise may provide a clinically important tool for the daily management of different aspects of several pathologies, offering an adapted solution to promote a suited program of physical activity for unhealthy subjects.

## 6. Isoinertial Training Technology: A Brief History Using the Isoinertial Method

### Highlight by James P. Fisher and Luke Carlson

In 1882, Adolf Fick [24] observed that a contracting muscle under stretch (e.g., lengthening; eccentric) could produce a greater force than a shortening muscle action (e.g., concentric) [25]. However, is was nearly 100 years later that this appeared to gain application in the area of resistance training, with a view of muscular adaptations. In the 1970s, Dr. Paavo Komi is alleged to have trained a group of Scandinavian weightlifters by having them lower—not lift—supramaximal loads (e.g., >1-repetition maximum—referred to as accentuated eccentric loading (AEL)). Certainly, Komi is an early name in the research surrounding eccentric and concentric strength training as well as increases in muscle strength [26]. 

Around the same time, Arthur Jones in the USA was also training a number of athletes using eccentric only training, but was reportedly frustrated with the impractical need for multiple trainers to repeatedly lift a load for a trainee to then lower the load for repetitions. Borne of this inefficiency, Jones developed a line of upper body Nautilus machines called the Super Omni. These resistance machines allowed the user to perform (or assist with) the concentric phase of an exercise by using a foot pedal and leg press mechanism. In turn, following the release of the foot pedal, this permitted the performance of the eccentric component of the exercise using a heavier load than could have been lifted traditionally. In part research and part promotion for Nautilus, Casey Viator (1971 Mr. America) used many of these machines for four weeks of almost exclusively eccentric only training in the Colorado Experiment, through May of 1973. Following a period of detraining and illness resulting from an anti-tetanus allergic reaction, and under the overview of Dr. Elliot Plese and supervision of Jones for each workout, he reportedly regained ~63 lbs of muscle in 28 days [27]. Notably, lower body exercises that could not be completed using the same process were performed using negative accentuated methods, whereby a trainee lifts a load bilaterally and lowers the load unilaterally (e.g., for a knee extension exercise, the concentric phase is performed with both limbs, and then one limb is relaxed while the other limb performs a controlled eccentric muscle action, lowering the load to the start position). This process effectively serves to double the resistance for the eccentric phase of an exercise. Jones sold Nautilus in 1986, and his subsequent exercise company, MedX, didn not produce a line of resistance machines where eccentric only training could be performed without the aid of trainers to assist in lifting the load. It is believed the expense of the Super Omni machines and the lack of uptake from a commercial perspective ended their production. 

Eccentric only, or accentuated eccentric load training (AEL), has moved in multiple directions since that time. A number of studies have reported the use of weight releasers, which attach to a barbell to add load to the eccentric phase, and upon reaching the lowest point in the range of motion, rest on the floor and detach [28,29]. However, while this system can be useful when performing single repetitions, their application is limited, as a set of repetitions is not practical with this tool. Another increasingly popular method of applying an eccentric overload is that of flywheel inertia training (FIT), where concentric muscle actions serve to unravel a cable or band rotating a flywheel. As the band reaches its maximum length, the flywheel continues to spin and winds the band on to the shaft again, requiring eccentric muscle action to slow the kinetic energy of the flywheel. In this sense, the more explosive the concentric muscle action, the faster the flywheel rotates, and the more force is applied through the cable for the eccentric muscle action. In review, flywheel training has shown positive increases in muscular strength, hypertrophy, and power [30], and due to the absence of a weight stack, has been recognised for its potential application in space travel and micro-gravity environments [31].

In addition to the growing areas of technology, two methods that are notable for their exclusion in recent reviews around eccentric training [32,33] rely on more humble approaches, namely: those of Nordic hamstring curls and manual/partner applied resistance. Nordic hamstring curls are performed by kneeling on a pad and anchoring the ankles by a partner or other immovable object, and then lowering the body under control from the knee, keeping the hips in extension. They have regained popularity over the last decade or so for their claimed injury prevention benefits for the avoidance of hamstring injury in soccer players [34], but rely predominantly on the upper body mass of the trainee. In contrast, manual applied resistance uses a partner to apply force against a limb to put the corresponding muscle(s) under tension. Whilst the Nordic hamstring exercise has grown in popularity, manual applied resistance seems to have diminished in areas other than personal training. The technique is credited to have been refined and popularized by Dan Riley, former college and NFL strength and conditioning coach, and is detailed in his book [35]. Academic support is given by Hedrick [36], and Dorgo and colleagues [37]. While manual applied resistance training is not necessarily known for eccentric training, Hedrick [36] states an advantage as the ability to adjust resistance throughout the range of motion of an exercise, based on the strength curve. In this sense, and in application, a partner can relatively easilily apply a greater force, and thus accentuate the eccentric overload through the negative phase of an exercise. 

The most recent developments in eccentric training are those of X-Force resistance machines (released in 2009), which achieve an eccentric overload by tilting the weight stack through 45° for the concentric phase, and then rotating the weight stack back to vertical for the eccentric phase. The manufacturers claim that this achieves a 40% increase in load for the eccentric phase, and produce 14 selectorized machines that are designed to replace traditional resistance machines in their application. The manufacturers promote a 3–1–5 s repetition duration (concentric–isometric–eccentric), allowing for a pause at the end of the concentric movement for the weight stack to tilt to vertical, and an accentuated 5 s eccentric phase to lower the increased weight under control. However, limitations include the cost of these pieces compared with traditional resistance machines, and the use of electrical power in the tilting of the weight stack, which might be high when using multiple machines across a fitness facility [38]. Furthermore, to date, no empirical research seems to have evaluated the efficacy of X-Force resistance machines for physiological responses to training.

It is apparent that eccentric exercise using isoinertial methods seems to undulate in and out of fashion, and has perhaps fluctuated in interest with the development of technology. At worst, the methods discussed herein might be acknowledged for their versatility (manual resistance training) or novelty, and thus the ability to remove boredom and stagnation from repetitive resistance training programmes. Other subsections within this Journal Club will review the effectiveness of eccentric training using isoinertial methods. 

## 7. Specific and General Eccentric Strength Adaptations

### Highlight by James P. Fisher

Resistance training and corresponding strength adaptations appear to be optimised by developing the motor schema of both technical elements of an exercise, in combination with the synchronous recruitment of motor units [39,40]. However, in some cases, a desirable outcome from strength training is not the improvement in the strength of specific exercises/movements, but rather the improved functioning across multiple movements, and in daily life, by a more general increase in the strength of a muscle or muscle group. This might be key in strength acquisition transfer to other meaningful areas, such as rate of force development, velocity, and power [41], although how well these translate to enhanced sports performance is still an area of debate [42]. 

In considering accentuated eccentric loading using isoinertial resistance training, we also see the transfer of force production to other contraction types [43]. However, the data has produced equivocal results as to the efficacy of accentuated eccentric loading/elongated time for eccentric muscle actions, and there remains a dearth of literature considering multi-joint, isoinertial exercises. For example, Walker and colleagues (2016) [43] reported greater eccentric and isometric strength increases for persons training with a 40% greater load for the eccentric phase of a repetition (but still performing both concentric and eccentric phases) compared with traditional training. In contrast, Fisher and colleagues (2016) [44] reported similar concentric strength increases between three groups, namely: an eccentric only group that trained using a 30% greater load and performing eccentric only repetitions (10 s), a group training using a traditional load but elongating the eccentric phase of the repetition (2 s concentric: 10 s eccentric), and a control group performing traditional repetitions (2 s concentric: 4 s eccentric) to momentary failure. The data has been the best presented in recent reviews [32,45], where it seems that, in summary, both concentric and eccentric muscle actions should be performed, and that the intensity of the effort remains a key variable (as it is in traditional resistance training). However, it is clear that eccentric training transfers to strength increases for other muscle actions, but is specifically important for eccentric strength [46]. More so, eccentric accentuated training appears to incur greater adaptations in eccentric strength compared with traditional training [43]. Evidence suggests that these strength increases are aligned with greater motor unit recruitment. For example, in the study by Walker and colleagues (2016), the favourable increases in isometric and eccentric strength for the accentuated eccentric training group were accompanied by increases in muscle activation.

In practical terms, improved eccentric strength, specifically in the hamstrings muscle group by use of movements such as the Nordic hamstring exercise, appear beneficial in reducing injury risk [34]. Furthermore, based on the necessity for eccentric strength for decelerating forces, including normal locomotion and change of direction, as well as in the storage of elastic recoil energy [25,47], future research should focus upon adaptations attained by using practical approaches to eccentric training using the isoinertial method.

## 8. The Undiscovered Benefits of Isoinertial Resistance Training under Unstable Conditions

### Highlight by Cristina Cortis and Andrea Fusco

The benefits of eccentric contractions, when compared with other contraction modalities, include the generation of greater maximal force/tension, a lower metabolic cost for the same work produced, more effectiveness in stimulating gains in muscle strength, and regional hypertrophy and muscle architectural remodeling [48]. As eccentric muscle strength declines with age to a lesser degree than other types of muscle strength, it may represent an effective stimulus for adaptation in older adults, playing a key role in successful ageing [49,50,51]. In particular, isoinertial resistance exercise has been suggested as a potential training modality in certain aging populations ready for higher resistance exercise stress [52,53], with studies mainly focusing on knee extension [53,54,55] and squat [56,57] exercises.

Flywheel knee extension trainings (8–12 weeks; 3 times/week; 7–12 repetitions; 1–4 sets per session) elicited beneficial modifications in muscle mass, strength, and architecture, and improved the quality of life and functional performances in 68-year old men [53,55] and the peak knee extension power in 70-year old men and women [54]. Isoinertial squat trainings (6–8 weeks; 2–3 times/week; 9–15 repetitions; 2–4 sets per session) improved mobility, balance, mean concentric propulsive velocity, and power in 65-year old men and women [57], as well as physical and functional abilities in 68-year old women [56]. While strength-related gains could be expected after isoinertial resistance trainings, improvements in functional abilities, specifically balance, are quite intriguing. In particular, the squat exercise, used in some studies [56,57], mirroring the sit-to-stand movement, could be one of the best isoinertial modality to perform in older adults, also being functional for activities of daily living. Isoinertial training could be responsible for a greater activation in hip and knee extensions, which made the older adults rely more on hip strategy when recovering from postural imbalance [57], for the increased tendon stiffness in the gastrocnemius, and for the higher degree of transfer effect of knee extensors loading on the ankle plantar flexors, improving balance performances [54].

Based on the transfer of the training adaptations principle, the concurrent presence of resistance exercises and instability might provide a more effective transfer of functional adaptations needed during the activities of daily living [58]. Although unstable surfaces and unbalanced conditions (i.e., Swiss balls, inflated discs, wobble boards, and suspension slings) have been mainly used for dynamic balance assessment and training in healthy, injured, and old subjects [59,60,61,62,63,64], they have been recently used also for strength gains purposes [58,65]. Despite extensive literature existing on the impact of instability resistance training on balance, stability, and strength [64,65], no experimental data are available on the functional training adaptations associated with isoinertial resistance training under unstable conditions in older populations. Therefore, future research should investigate the functional gains associated with the combined effect of the isoinertial and instability resistance training in order to have successful ageing.

## 9. The Drop Jump and Accentuated Eccentric Loading

### Highlight by Lee A. Bridgeman

In recent years, there has been an increase in the interest of the use of eccentric training, with several reviews dedicated to exploring this training modality [32,33,45,66,67]. An eccentric muscular action involves the lengthening of a muscle as a result of an external load [29,50,68]. During a concentric muscle action, the actin and myosin detachment is ATP-dependent, whereas an eccentric muscle action occurs as a result of a mechanical detachment of active cross-bridges [29,69]. The mechanical nature of eccentric muscle actions has been suggested to result in a greater force production. There are also reported to be significant differences in neural activation between concentric and eccentric muscle actions [70]. Typically, loading during resistance training has been the same during both the concentric and eccentric portion of the exercise, despite the finding that athletes could be up to 50% stronger during the eccentric phase. Therefore, this may represent a missed opportunity to train optimally in order to enhance athletic performance. One training method that seeks to address this issue is accentuated eccentric loading (AEL). This method includes the use of greater loads during the eccentric phase, a coupled eccentric and concentric movement, and does not change the nature of the movement. One training activity that seems to fit the aforementioned criteria is the use of a drop jump (DJ) with an AEL, which allows the concentric phase to be completed as normal, without greatly altering the movement. In a study that investigated five DJs with no additional load, and 10%, 20%, and 30% AEL using dumbbells to provide the eccentric overload, it was reported that an AEL equivalent to 20% of a subject’s body mass resulted in enhanced subsequent countermovement jump (CMJ) performance two and six minutes after the initial DJs [70]. This was suggested to be the result of the AEL utilised during the DJs, resulting in a greater activation of Type II motor units, and therefore the greater potentiation of these motor units in the subsequent CMJs. Wagle et al. [45] propose that these results not only indicate an optimal eccentric load exists, but also there is an optimal time period to take advantage of any potentiation effect. Therefore, further research is required in order to examine the acute effects in greater detail, and also to see if AEL DJs, when included as part of a longer term training programme, can enhance jump performance.
